# A multilevel analysis of effect of neighbourhood and individual wealth status on sexual behaviour among women: evidence from Nigeria 2003 Demographic and Health Survey

**DOI:** 10.1186/1472-698X-8-9

**Published:** 2008-06-27

**Authors:** Olalekan A Uthman, Eugene J Kongnyuy

**Affiliations:** 1 Center for Evidence-Based Global Health, Save the Youth Initiative, Nigeria; 2 Child and Reproductive Health Group, Liverpool School of Tropical Medicine, Liverpool, UK

## Abstract

**Background:**

Nigeria is home to more people living with HIV than any other country in the world, except South Africa and India – where an estimated 2.9 million [1.7 million – 4.2 million] people were living with the virus in 2005. Women bear the greatest burden of frequent high-risk pregnancies, raising large families, and increasingly, the AIDS epidemic. Thus, there is a need for better understanding of the determinants of high risk sexual behaviour among women. In this study, we examined factors associated with extra-marital sex among women in Nigeria and investigated how much variation in reported extra-marital sex can be attributed to individual-, and community-level factors.

**Methods:**

We analyzed data from 6362 sexually active women aged 15 – 49 years who participated in the Nigeria 2003 Demographic and Health Survey using multilevel logistic regression models. Results are presented as odds ratio with 95% confidence interval.

**Results:**

Independent of other factors, compared to women aged 15–24 years, those 25 – 34 years (odds ratio [OR] 0.59; 95% CI: 0.44 – 0.79) and 35 years or older (OR 0.36; 95% CI: 0.24 – 0.54) were less likely to have reported multiple concurrent sex partners in the last 12 years. As expected, women currently or formerly married were less likely to have reported multiple concurrent sex partners than women never married. Women who drank alcohol in the last three months were more likely to have reported multiple concurrent sex partners. Compared to women from richest household, women from poorest and middle household were 83% and 51% more likely to multiple concurrent sex partners in the last 12 month respectively. After individual compositional and contextual factors, community wealth status was statistically significant with sexual behaviour.

**Conclusion:**

The study has demonstrated that individual and community wealth status are independent predictors of women's sexual behaviour, and that there is significant neighbourhood variation in odds of multiple concurrent sex partners, even after controlling for effects of both individual- and community-level characteristics. Scholars trying to understand variation individual high risk sexual behaviour should pay attention to the characteristics of both individuals and places of residence.

## Background

Nigeria is home to more people living with HIV than any other country in the world, except South Africa and India – where an estimated 2.9 million [1.7 million – 4.2 million] people were living with the virus in 2005 [1]. As is the case in most of sub-Saharan Africa, women greatly surpass men in the number of people living with HIV/AIDS, and in many areas women double the number of men with the virus [1]. Reasons for women's increased risks include biological and social risk factors [2]. The vulnerability that stems from poverty and limited job opportunities often means that women do things that they would rather not. Exposure to multiple partners increases the risks for sexual transmitted infections (STIs) and STIs facilitate the transmission of human immunodeficiency virus (HIV) [3,4].

In the context of Sub-Saharan Africa countries, existing studies on association between wealth and high risk sexual behaviour often focus exclusively on males [5-7]. Women bear the greatest burden of frequent high-risk pregnancies, raising large families, and, increasingly, the AIDS epidemic. Together, these conditions have had devastating consequences for the health and well-being not only of African women but also their families. It is increasingly clear from the research that young women in sub-Saharan Africa are at particularly high risk of HIV infection. There is some evidence that the place where a person lives may not affect all people in the same way [8-11]. Recent research suggests that the neighbourhood environment is more important for women than for men [12-14]. The social environment appears to be particularly important for women's health status. Thus, there is a need for better understanding of the determinants of high risk sexual behaviour among women.

In this study, we examined the individual- and community-level socioeconomic, demographic, and behavioural predictors of extra-marital sex among women in Nigeria. We additionally examine whether there is significant neighbourhood variation in extramarital sex and neighbourhood variation is explained by health risk factors at the individual-level and community-level.

## Methods

### Study design

Cross-sectional and population-based study using data from the 2003 Nigeria Demographic and Health survey (NDHS).

### Sampling technique

Methods used in the NDHS have been published elsewhere [15]. Briefly, the survey used a two-stage cluster sampling technique. The country was stratified into 36 states and the Federal Capital Territory (FCT) of Abuja. Each domain is made up of enumeration areas (EAs) established by a general population and housing census in 1991. The sampling frame was a list of all EAs (clusters). Within each domain, a two-stage sample was selected. The first stage involved selecting 466 clusters (primary sampling units) with a probability proportional to the size, the size being the number of households in the cluster. The second stage involved the systematic sampling of households from the selected clusters. A nationally representative probability sample of 7864 households was then selected from the clusters, in which all women aged 15 to 49 years were eligible to be interviewed.

### Data collection

Data collection procedures have been published elsewhere [15]. Briefly, data were collected by visiting households and conducting face-to-face interviews to obtain information on demographic characteristics, wealth, anthropometry, female genital cutting, HIV knowledge, and sexual behaviour between March and August 2003.

### Ethical consideration

This study is based on an analysis of existing survey data with all identifier information removed. The survey was approved by the Ethics Committee of the ORC Macro at Calverton in the USA and by the National Ethics Committee in the Ministry of Health in Nigeria. All study participants gave informed consent before participation and all information was collected confidentially.

### Variables

#### Outcome variable

For the present study, 6362 never, currently, or formerly married women, all of whom who have had at least one episode of sexual intercourse in their lifetime, were drawn from the overall sample of 7620 women to examine factors associated with "high-risk" sexual behaviour. Thus, if a woman has never had sexual intercourse in her life (e.g., a "virgin"), she was not included in the analysis. "High-risk" sexual behaviour was defined as having two or more sex partners in the last 12 months.

#### Explanatory variables

The correlates of sexual behaviour were selected by reviewing the literature and grouped into individual- and community-level factors.

##### Individual-level factors

*Age *was categorized into three groups: 15–24 years, 25–34 years, and 35 years or older.

*Education attainment *was grouped into three bands: never been to school, primary, and secondary or higher education.

*Wealth index *a score was attributed to each household amenity and the total score constituted the wealth index score. We divided this score into three classes of wealth: poorest (below 20th quantile), middle (between 20th and 80th quantile), and richest (above 80th quantile).

*Past alcohol use*: past alcohol use was defined as number of days drank alcohol last three months (never, one day, two days, three or more days)

*Marital status: *grouped into never, currently, or formerly married.

*Religion*: respondents' religion was stratified into Christian, Muslim, and others

#### Community-level factors

*Community economic status *is an average wealth index at weight index at community level. Economic inequality is measured by dividing community wealth index into three equal quintiles.

*Place of residence *was defined as rural or urban

*Geographic region *1) North central, 2) North East, 3) North west, 4) South East, 5) South south, and 6) South west

### Statistical analyses

The descriptive statistics show the distribution of respondents by the key variables. Values were expressed as absolute number (percentages) and mean (standard deviation) for categorical and continuous variables respectively.

Given the hierarchical structure of the sample and the binary outcome, a logistic multilevel modelling approach was adopted. A two-level model with a binary response (*y*, whether the respondent had extramarital sex in the past 12 months or not) for a woman *i *living in community *j *of the form:

(1) *π*_*ijk*_: *y*_*ijk*_~Bernoulli(1, *π*_*ijk*_)

The probability was related to a set of categorical predictors, *X*; and a random effect for each level, by a logit-link function as

(2) logit(*π*_*ij*_) = log [*π*_*ij*_/(1 - *π*_*ij*_)] = *β*_0 _+ *βX*_*ij *_+ u_0*j*_

The linear predictor on the right-hand side of the equation consisted of a fixed part (*β*_0 _+ *β X*_*ij*_) estimating the conditional coefficients for the explanatory variables, and random intercept attributable to communities (*u*_0*j*_).

The analysis was done in three steps. In Model 1 (empty model), no explanatory variable was included. In model 2, only individual-level factors were included. Model 3 we controlled for both individual and community-level factors. The results of fixed effects (measures of association) were shown as odds ratios (ORs) with 95% confidence intervals (CIs). The results of random effects (measures of variation) were presented as variance partition coefficient and percentage change in variance.

The MLwiN software, version 2.0.2, was used for the analyses. Parameters were estimated using the Markov Chain Monte Carlo (MCMC) procedure. The default settings in MLwiN were used for the analyses, i.e., chains of length 5000 after a burn-in of 500. The Deviance Information Criterion (DIC) was used as a measure of how well our different models fitted the data. A lower value on DIC indicates a better fit of the model.

## Results

### Sample characteristics and unadjusted associations

A total of 6362 sexually active women nested within 177 communities participated in the study (Table 1).

**Table 1:** Socio-demographic characteristics of sexually active Nigeria women who participated in the study, 2003

***Variable***	***Number of women (%)***	***Percent of women who reported multiple concurrent sexual partners in the last 12 months (%)***
**Individual covariates**		
**Age groups**		
15 – 24	2009 (31.6)	28.1
25 – 34	2251 (35.4)	8.7
> 35	2102 (33.0)	3.2
**Level of education**		
No school	2809 (45.5)	2.9
Primary	1387 (21.8)	9.2
Secondary/higher	2081 (32.7)	29.5
**Wealth index**		
Low	1350 (21.3)	8.0
Middle	3799 (59.7)	12.4
High	1211 (19.0)	20.3
**Past alcohol use**		
No	5692 (89.7)	11.3
1	146 (2.3)	32.2
2	111 (1.8)	26.1
3+	396 (6.2)	26.8
**Marital status**		
Never married	831 (13.1)	77.4
Currently married	5156 (81.0)	1.8
Formerly married	375 (5.9)	25.1
**Religion**		
Christian	2995 (47.1)	22.8
Muslim	3245 (51.0)	4.2
Others	118 (1.9)	6.8
**Community covariates**		
**Community economic status**		
Low	2238 (35.2)	7.4
Middle	2126 (33.4)	13.0
High	1998 (31.4)	19.3
**Place of residence**		
Urban	2483 (39.0)	16.2
Rural	3879 (61.0)	10.9
**Region**		
North central	1048 (16.5)	13.4
North East	1256 (19.7)	5.8
North west	1650 (25.9)	2.1
South East	765 (12.0)	22.4
South south	781 (12.3)	33.3
South west	862 (13.6)	17.2

Respondents were fairly evenly distributed across the age groups, 32% were aged 15–24, 35% were 25–34 years, and 33% were 35 or older. Almost half (46%) of the women were had no education. Majority of the women were currently married (82%) and 6% were formerly married. Table 1 also present unadjusted univariable associations between sexual behaviour and each explanatory variable. Using cross-tabulations and Pearson's chi-squared test, all explanatory variables were strongly related to sexual behaviour at p < .0001.

### Multilevel analysis

The result of the random effects model is shown in Table 2 (Empty Model 1). There is significant variation in the log odds of reporting multiple concurrent sexual partners in the last 12 months the communities (*τ *= 1.50, *p *= .001). According to the intra-community correlation coefficient implied by the estimated intercept component variance, 31% variance in the odds of reporting multiple concurrent sexual partners could be attributed to community-level. This variation remained significant, even after controlling for individual-level factors (Model 2) and both individual and community-level characteristics (Model 3). As judged by proportional change in variance, 66% and 74% of the variance in the log odds of reporting multiple concurrent sexual partners variance across communities was explained by individual compositional factors (Model 2) and both individual compositional and contextual factors (Model 3) respectively. In addition, the deviance information criterion (DIC) – was significant to reveal that the individual compositional factors (Model 2) and individual compositional and contextual variables (Model 3) increased the multivariable multilevel model's ability to explain variation in the log odds of reporting multiple concurrent sexual partners, as indicated by lower DIC.

**Table 2:** Individual compositional and community contextual factors associated with sexual behaviour among Nigerian women identified by multivariable multilevel logistic regression

	***Empty model***	***Model with individual variables***	***Model with individual and community variables***
	
	**Model 1**	**Model 2**	**Model 3**
	
	**OR (95% CI)**	**OR (95% CI)**	**OR (95% CI)**
***Individual variables***			
**Age groups**			
15 – 24		reference	reference
25 – 34		0.57 (0.43, 0.77)***	0.59 (0.44, 0.79)***
> 35		0.35 (0.24, 0.53)***	0.36 (0.24, 0.54)***
**Level of education**			
No school		reference	reference
Primary		0.97 (0.64, 1.48)	0.88 (0.57, 1.36)
Secondary/higher		1.63 (1.06, 2.50)*	1.53 (0.99, 2.38)
**Wealth index**			
Low		1.83 (1.14, 2.94)*	1.95 (1.08, 3.50)*
Middle		1.51 (1.07, 2.12)*	1.69 (1.14, 2.51)**
High		reference	reference
**Past alcohol use (days)**			
No		reference	reference
1		2.61 (1.35, 5.06)**	2.70 (1.41, 5.17)**
2		4.69 (2.27, 9.69)***	4.42 (2.14, 9.13)***
3+		3.83 (2.49, 5.89)***	3.47 (2.25, 5.34)***
**Marital status**			
Never married		reference	reference
Currently married		0.05 (0.01, 0.09)***	0.05 (0.01, 0.09)***
Formerly married		0.16 (0.10, 0.23)***	0.15 (0.10, 0.23)***
**Religion**			
Christian		reference	reference
Muslim		0.58 (0.41, 0.83)**	0.72 (0.49, 1.07)
Others		0.54 (0.19, 1.51)	0.52 (0.19, 1.46)
**Community covariates**			
**Community economic status**			
Low			0.90 (0.53, 0.97)*
Middle			0.73 (0.48, 0.87)**
High			reference
**Place of residence**			
Rural			1.01 (0.71, 1.44)
Urban			reference
**Region**			
North central			reference
North East			1.08 (0.63, 1.83)
North west			0.44 (0.24, 0.81)*
South East			0.71 (0.44, 1.17)
South south			1.56 (0.97, 2.51)
South west			0.76 (0.47, 1.24)
Intercept	0.10 (0.09, 0.12)***	2.12 (1.22, 3.71)**	2.40 (1.11, 5.17)*
**Random effects**			
Community random variance (SE)	1.51 (0.20)***	0.51 (0.14)***	0.39 (0.13)**
Variance partition coefficient (%)	31.4	13.4	10.5
Explained variance (%)	reference	66.3	74.3
**Model fit statistics**			
DIC	4366	2050	2044

Abbreviations: OR – Odds ratio, CI – Confidence interval, SE – Standard error, DIC – Deviance information criterion

*p < .05, **p < .01, and ***p < .001

The results of fitting the model including individual-level variables appear in Table 2 (Model 2). The effect of the inclusion of individual- and contextual factors is shown in Table 2 (model 3). Inclusion of the community-level variables had minimal effect on the contribution of individual-level variables to the likelihood of reporting multiple concurrent sexual partners in the last 12 months. After individual compositional and contextual factors, the effect of age, wealth status, past alcohol, and marital status remained significant. Independent of other factors, compared to women aged 15–24 years, those 25 – 34 years (odds ratio [OR] 0.59; 95% CI: 0.44 – 0.79) and 35 years or older (OR 0.36; 95% CI: 0.24 – 0.54) were less likely to reported multiple concurrent sex partners. As expected, women currently or formerly married were less likely to have reported multiple concurrent sex partners in the last 12 months than women never married. Women who drank alcohol in the last three months were more likely to have multiple concurrent sex partners in the last 12 months. Compared to women from richest household, women from poorest and middle household were 83% and 51% more likely to multiple concurrent sex partners in the last 12 month respectively. After individual compositional and contextual factors, community wealth status was statistically significant with sexual behaviour.

## Discussion

This study examined the association between individual and community wealth status and sexual behaviour among resident Nigerian women using multilevel statistical framework. Our contention that wealth is associated with women's sexual behaviour was confirmed. This finding is in contrast to previous work [5,6] and consistent with those of others among men [7]. With underlying factors controlled for, wealthier women were less likely to have reported multiple concurrent sexual partners in the last 12 months. Contrary to previous studies [16,17], we found that increasing is negatively associated odds of reporting multiple sexual partners. This study has also provided evidence of positive association between past alcohol use and sexual behaviour. This is consistent with previous study that has examined this association among men [18].

Of particular interest in this investigation are possible effects of neighbourhood context on odds of reporting multiple concurrent sexual partners in the last 12 months. Neighbourhoods constitute a key determinant of socioeconomic disparities in health, as they shape individual opportunities and expose residents to multiple risks and resources over the life course. Using multilevel framework, this study that has shown that both individual-level and community-level characteristics are important predictors of sexual behaviour (as measured by multiple concurrent sexual partners) in Nigeria, and demonstrates significant neighbourhood variation in odds of reporting multiple concurrent sexual partners. The individual- and community-level characteristics included in the model are able about three-quarter (74%) of these observed variations. These findings have important implications for targeting policy as well as the search for left-out variables that might account for this unexplained variation.

### Study limitations and strengths

There are a number of caveats to be considered when interpreting these results. The cross-sectional nature of the data limits ability to draw casual inferences. We were unable to take into account the impact of residential changes over time and the cumulative effects of socioeconomic environment over time. Another limitation of this study worth mentioning is that measuring wealth is problematic. The study can be criticized for using an indirect measure of household wealth. However, due to the fact that in developing countries like Nigeria it is hard to obtain reliable income and expenditure data, an asset-based index is generally considered a good proxy for household wealth status. Many of the household wealth indices use assets that are more likely to be found in urban areas than in rural areas. Thus, most of the rural households will be in the lowest wealth category even if they have other indicators of wealth (e.g., livestock or farm machinery). Another important limitation is validity constraints of self-reported sexual activity. By definition, self-reports of initiation of sexual activity cannot be externally validated and studies have revealed considerable inconsistencies in individual's self-reported sexual activity [19,20]. There is evidence that women tend to underreport their premarital and extramarital sexual activity [21]. Despite these limitations, the study strength is significant. It is a large, population-based study with national coverage.

## Conclusion

Using an explicit multilevel analytic framework, the study has demonstrated that individual and community wealth status are independent predictors of women's sexual behaviour, and that there is significant neighbourhood variation in odds of reporting multiple concurrent sexual partners, even after controlling for effects of both individual- and community-level characteristics. Future studies should investigate other factors that may account for the unexplained neighbourhood variation. Future research also should address the mechanisms that connect the individual and neighbourhood levels. Although this study does not investigate these mechanisms, the findings clearly provide evidence that social context is associated with sexual behaviour independent of individual-level factors, challenging a purely individualistic approach to sexual behaviour, and pointing to the importance of health promotion and prevention at the community level. Scholars trying to understand variation individual sexual behaviour should pay attention to the characteristics of both individuals and places of residence.

## Competing interests

The authors declare that they have no competing interests.

## Authors' contributions

OAU conceived the study, extracted the data, did the analyses and interpretation, and wrote the first draft of the manuscript. EJK participated in the interpretation and critically revised the manuscript for important intellectual content. Both authors read and approved the final manuscript.

## Pre-publication history

The pre-publication history for this paper can be accessed here:


